# From wings to whiskers to stem cells: why every model matters in fragile X syndrome research

**DOI:** 10.1186/s11689-024-09545-w

**Published:** 2024-06-13

**Authors:** Soraya O. Sandoval, Natasha M. Méndez-Albelo, Zhiyan Xu, Xinyu Zhao

**Affiliations:** 1 Waisman Center, University of Wisconsin-Madison, Madison, WI 53705 USA; 2 Department of Neuroscience, School of Medicine and Public Health, University of Wisconsin-Madison, Madison, WI 53705 USA; 3 Neuroscience Training Program, University of Wisconsin-Madison, Madison, WI 53705 USA; 4 Molecular Cellular Pharmacology Training Program, University of Wisconsin-Madison, Madison, WI 53705 USA; 5 Graduate Program in Cell and Molecular Biology, University of Wisconsin-Madison, Madison, WI 53705 USA

**Keywords:** Fragile X syndrome, FMR1, Human, iPSCs, Stem cells, Neuron, Organoid, Mouse, Drosophila, FMRP

## Abstract

Fragile X syndrome (FXS) is caused by epigenetic silencing of the X-linked fragile X messenger ribonucleoprotein 1 (*FMR1)* gene located on chromosome Xq27.3, which leads to the loss of its protein product, fragile X messenger ribonucleoprotein (FMRP). It is the most prevalent inherited form of intellectual disability and the highest single genetic cause of autism. Since the discovery of the genetic basis of FXS, extensive studies using animal models and human pluripotent stem cells have unveiled the functions of FMRP and mechanisms underlying FXS. However, clinical trials have not yielded successful treatment. Here we review what we have learned from commonly used models for FXS, potential limitations of these models, and recommendations for future steps.

**Supplementary Information:**

The online version contains supplementary material available at 10.1186/s11689-024-09545-w.

## Background

Fragile X syndrome (FXS) is the most inherited form of intellectual disability and autism [1]. It is caused by epigenetic silencing of the X-linked fragile X messenger ribonucleoprotein 1 (*FMR1)* gene located on chromosome Xq27.3, which leads to the loss of its protein product, fragile X messenger ribonucleoprotein (FMRP) [2–4]. FXS has a higher penetrance in male than female patients, with a prevalence of 1 in 4000 males and 1 in 7000 females [5, 6]. The 5’ untranslated region (UTR) of the human *FMR1* gene has polymorphic CGG trinucleotide repeats, with 30 repeats as the mode in the human population [7, 8]. The expansion of 55 to 200 repeats is associated with pathological conditions, including neurodegenerative disorder fragile X-associated tremor/ataxia syndrome (FXTAS) in males and fragile-X-associated primary ovarian insufficiency (FXPOI) in females [9]. The expansion of CGG repeats to over 200 leads to DNA methylation and silencing of the *FMR1* gene, which is the main cause of FXS. However, there are also FXS patients who do not have a CGG repeat expansion but have rare *FMR1* coding region mutations that lead to a loss of FMRP function [10].

FMRP is an RNA binding protein that can regulate gene expression through multiple mechanisms, such as protein translation, mRNA stability, RNA transport, and chromatin remodeling [11]. FMRP is widely expressed in many tissue and cell types with the highest expression mainly expressed in the brain and the testes [12]. FMRP is mainly expressed in the cytoplasm but contains a nuclear localization signal (NLS) and a nuclear exportation signal (NES) [13], which allows it to enter the nucleus to carry pre-messenger ribonucleoprotein complexes (pre-mRNP) back to the cytoplasm to interact with polyribosomes and regulate protein translation in neurons. FMRP also has two Tudor domains (TD1 and TD2) [14–16] involved in protein-protein interactions and DNA binding, and three KH domains (KH0, KH1, and KH2) and one RGG box for RNA-binding [7, 11, 17]. Since the discovery of *FMR1* as the gene responsible for FXS, much effort has been devoted to identifying FMRP regulated mRNAs and molecular pathways as therapeutic avenues to treat FXS [18–21]. It is known that FMRP and its targets regulate important processes of neurodevelopment, such as synaptogenesis, neurogenesis, and cytoskeleton formation, which share significant overlap with pathways implicated in autism [11, 22].

FXS patients may exhibit a wide range of symptoms, with the most common being moderate to severe intellectual disability, language impairment, anxiety, hyperactivity, aggression, and increased seizure risk [1, 22]. They also have physical attributes such as elongated face, large ears, joint hypermobility, and macroorchidism [9]. Most patients have many behavioral symptoms that overlap with autism such as attention deficit disorder, repetitive behaviors, sleep problems, and sensory overload [9, 23]. To understand the pathophysiology of FXS, researchers have used genetic techniques to create animal models lacking FMRP expression. The most widely used models are the knockout mouse models (*Fmr1*-KO and *Fmr1*-floxed mice) and knockout drosophila models (d*Fmr1*-KO drosophila). In the last decade there has also been increasing research performed in neurons or organoids derived from human pluripotent stem cells (hPSCs) including both human induced pluripotent stem cells (hiPSCs) and human embryonic stem cells (hESCs). Several recent review articles have provided detailed descriptions about these models [24–27]. Here, we will compare the strengths and weaknesses among the major models of FXS: the d*Fmr1*-KO drosophila, *Fmr1*-KO rodent models, and human FXS stem cell models, and discuss how they complement each other to advance our understanding in FXS.

## Review

### Drosophila model of FXS

*Drosophila melanogaster* is one of the most useful model organisms in biology because its small genome and its rapid reproduction cycle allow for fast genetic manipulations to study many genetic disorders, including FXS [27]. The functional domains between human FMRP and *Drosophila* FMRP are highly conserved, with the KH1 and KH2 domains being 35% identical and 65% similar [27–29]. FMRP in *Drosophila* is highly localized to the brain and the eyes, especially in mushroom bodies which resemble the human hippocampus, important for learning and memory [26, 30, 31]. FMRP in drosophila has been shown to have important roles in synaptic plasticity [30, 31], regulation of calcium signaling [32, 33], apoptosis [34], phagocytosis [35], and regulation of circadian rhythm [36]. Like most models of FXS, the *D.melanogaster* FXS model was created by deleting the human *FMR1* homolog, *Fmr1* [31] and many cellular, molecular, and physiological deficits found in *Fmr1-*KO *Drosophila* overlap with those found in other animal models and the symptoms of FXS patients [26, 27, 37] (Table S1).

#### Behaviors of Drosophila FXS models

Many of the behavioral phenotypes found in the *D.melanogaster* FXS model have matched with that of FXS patients to some extent. For example, 23–46% of FXS patients have sleep disorders, with FXS patients typically sleeping less than the general population [38]. The *Fmr1*-KO *Drosophila* exhibit significant deficits in circadian rhythm [39, 40]. In addition, *Fmr1*-KO *Drosophila* also have increased sleeping time during both the day and night compared to its control counterparts. Subsequent studies have shown that FMRP may regulate circadian rhythm through a number of mechanisms including microRNA processing [41], expression of FMRP target collapsing response mediator protein (CRMP) [42], insulin signaling [43], and interaction of FMRP with protein Ataxin2 [44].

Other common behavioral problems among FXS patients include hyperactivity, repetitive behaviors, and deficits in learning and memory, which have also been found in autism patients [45]. The *Drosophila* FXS model has elevated persistent grooming behavior which resembles the hyperactivity and repetitive behaviors found in some FXS patients [46]. *Fmr1*-KO larva have hyperactive locomotion due to elevated bone morphogenetic protein type 2 receptor (BMPR2) pathway [47]. When assessing learning and memory in drosophila, several studies have used the courtship paradigm and classical conditioning method. During the courtship paradigm, male flies are trained by being put with an unreceptive female in which they should learn they will be rejected in any attempt to court in the future. The *Drosophila* FXS model demonstrated the ability to learn but they failed to recall what they have learned, indicative of memory deficits [39, 48]. A study looking into the climbing ability of the *Drosophila* FXS model found that, overall, they were poorer climbers compared to control, and their climbing ability significantly declined with age [49], similar to how their learning capability also declines with age. Likewise, it has been shown that FXS patients have a decrease in IQ scores with aging [50], suggesting that FMRP plays an important role in aging and not only neurodevelopment.

#### Molecular mechanisms of Drosophila FXS models

Studies using *Drosophila* have been the trail blazer to unveil mechanisms underlying FMRP function and FXS, and many of these findings are confirmed in mammalian models. For example, the first identified FMRP mRNA target is *Futsch*, a homolog of mammalian microtubule associated protein 1B (*MAP1B)*, which has been confirmed in subsequent mouse [18, 20, 31] and human [19, 51] studies. In addition, reduced production of cyclic AMP levels were initially discovered in platelets [52] and lymphocytes [53] isolated from FXS individuals and later confirmed in brain tissue of both *Fmr1*-KO *Drosophila* and *Fmr1*-KO mice [54]. This has led to further research on targeting phosphodiesterase (PDE) as a treatment in *Fmr1-*KO mouse models [55, 56] and finally an ongoing clinical trial using a PDE-4D inhibitor for FXS [57].

#### Limitations of the Drosophila FXS Model

While *Drosophila* has served as an ideal model to identify mRNA targets for FMRP, the biochemical pathways identified in this model may not directly translate to humans, because of genetic differences among species. *Drosophila Fmr1* has three homologs in mammals, *FMR1, FXR1*, and *FXR2*. Drosophila *Fmr1* likely carries out the functions of both *FMR1* and its autosomal two mammalian paralogs. In addition, similar to using other animal models, the behavioral phenotypes of *Drosophila* may not fully reflect cognitive deficits observed in FXS patients. Furthermore, there have been conflicting results in locomotor activities of *Fmr1*-KO *Drosophila*, with studies showing no significant change [39], reduced activity [40], or increased activity [58]. Despite these differences, some of the key phenotypes and pathways identified in *Drosophila* have been replicated in mammalian models of FXS and gone to clinical trials. Therefore, despite its limitations, *Drosophila* serves as an effective and efficient model to investigate many aspects of FXS.

### Rodent models of FXS

The first model of FXS was the *Fmr1*-KO mouse model created by deletion of exon 5 [59]. The coding sequence of the mouse *Fmr1* gene is 97% identical to that of the human *FMR1* gene [60]. The mouse models for FXS, including an additional *Fmr1*-KO mouse line [61], a conditional knockout model (cKO) [61, 62], a point-mutant of FMRP RNA binding domain mouse model [63] and a conditional restoration (cON) line [61, 64], have been useful in studying the roles of FMRP in neurodevelopment and developing therapeutic targets. More recently, *Fmr1*-KO rat models have been generated [65, 66] which have the advantage of bigger size, better social behavioral measurement and higher genetic similarity to humans, compared to mouse models. Both mice and rat FXS models have striking similarity to FXS phenotypes and have served to confirm results identified in the *Drosophila* model as well.

#### Behaviors of rodent FXS models

FXS rodent models have been vital in our understanding of the role of FMRP in regulating behaviors and have led to significant progress in identifying treatment targets for clinical trials. Hyperactivity and repetitive behaviors are among the common clinical features of FXS patients [23]. Several studies have shown that FMRP deficient mice and rats exhibit these features, whereas other studies did not observe these phenotypes [24, 67, 68]. Seizures occur in a subset of young FXS patients with 10–20% of FXS patients reporting they have seizures in their teens and then this prevalence falls by the age of 20 [69]. Spontaneous seizures have not been reported in FXS mouse models; however, when audiogenic stimuli were used, juvenile *Fmr1*-KO mice had increased audiogenic seizures and defects in the acoustic startle response, similar to FXS patients [70]. In addition, both *Fmr1*-KO mouse and rat models exhibit increased resting state gamma oscillations and decreased alpha oscillations in their EEG recordings [71–73], similar to what has been shown in FXS patients [74]. Another prominent phenotype in FXS patients is anxiety. While studies have shown elevated anxiety-like behaviors in mouse [75] and rat models [71], other studies found that KO mice seem to be less anxious in an elevated plus maze test and more dominant in an automated tube test than wild-type mice [76]. Similar to the *Drosophila* FXS model, FXS rodent models also have impairments in spatial memory and spatial learning, reduced social interaction, increased grooming behavior, and aberrant circadian rhythm (Table S1). Limited studies have investigated sex-specific phenotypes in *Fmr1*-KO rodent models, with one study finding no sex differences in behaviors [77], while others showing some differences in behaviors and EEG recording between male and female *Fmr1*-KO rodents [71, 78]. Although FXS affects both males and females, female FXS patients are heterozygote for *FMR1* gene mutation and exhibit significantly milder symptoms than male FXS patients. Therefore, most animal studies have used male models, which we focus on in this review.

#### Molecular mechanisms of rodent FXS models

The neurons in *Fmr1*-KO mice and rats have a higher density of dendritic spines and shorter long thin dendritic branches [79–82], consistent with those found in a limited number of human FXS postmortem brains [83, 84]. Many of the genetic pathways identified in FXS models support the conclusion that increased protein translation at the synapse impairs neuronal plasticity leading to behavioral deficits. These discoveries have led to several clinical trials [23, 85]. Among these discoveries is the mGluR theory, which suggests that FMRP regulates synaptic protein synthesis through metabotropic glutamate receptors (mGluR1 and mGluR5), muscarinic acetylcholine receptors, and Gq-linked receptors, leading to mTOR-dependent signaling pathways to increase protein synthesis [86]. Mouse and rat *Fmr1*-KO models exhibit increased production of synaptic proteins and high levels of AMPA receptor internalization leading to enhanced mGluR-dependent long-term depression (LTD) in the hippocampus [66, 87], impaired circuit formation, seizures, and behavioral deficits [82, 88]. In addition, several other signaling pathways identified in the *Drosophila* model have also been reported as dysregulated in *Fmr1-*KO mice, including mTOR, GSK3β, MMP9, PI3K, MAPK, and insulin pathways, which are all implicated in regulating protein translation at the synapses (reviewed by [1, 11, 23, 89]).

#### Limitations of rodent FXS models

A major limitation of rodent models is the significant differences between primate and rodent brains, particularly in the prefrontal cortex (PFC) where FMRP is highly expressed [90]. Primate PFC is significantly larger proportionally, much more complex compared to other species [91], and exhibits gene signatures unique to primates [92]. In addition, FXS animal models have *Fmr1* gene deletion rather than epigenetic silencing of *FMR1* as a result of CGG expansion observed in a majority of human FXS patients, therefore *Fmr1*-KO rodent models cannot fully model the genetic complexity in human FXS including CGG expansion and retraction, DNA demethylation or loss of CGG repeats leading to *FMR1* gene reactivation, and somatic mosaicism [93, 94]. Several studies have attempted to model FXS CGG expansions and epigenetic silencing in mice but found that even with 300 human CGG repeats inserted into the 5’UTR of the mouse *Fmr1*, the CGG repeat containing mouse *Fmr1* gene failed to undergo DNA methylation and gene silencing [93, 95, 96]. Therefore, the transcriptional silencing that occurs in humans has not been replicated in mice.

Fortunately, despite differences among human and animal models, rodent models of FXS have been able to replicate behavioral and cellular changes seen in FXS patients. Some contradicting behavioral results from rodent studies might be due to many factors, including differences in genetic background of mice and methods of analyses. It is important to note that the same strain of mice might have sub strains that are phenotypically different from one another such as the C57BL/6J versus C57BL/6JN mice [97]. Therefore, it is crucial to select and report the appropriate mouse strains when assessing behavioral phenotypes. Another major contributing factor to the variable results of behavioral assessment is the methods used for behavioral testing among different laboratories, which has been a hot topic in the field with the goal to enhance reproducibility [24]. Overall, mice are a great model to understand the function of FMRP and to assess potential treatments for FXS in an in vivo system.

### Human stem cell models of FXS

#### hiPSC and isogenic hPSCs

HPSCs, including hiPSCs and hESCs, have become an important model for studying human brain development and neurodevelopmental disorders [11]. hiPSCs allow us to study molecular pathways in disease-relevant human cells derived from patients, facilitating drug screening for a more rapid transition of treatments to clinical trials. hPSCs and hPSC derived NPCs and 2-dimensional (2D) neurons have been used to develop new drug screens for FXS [98, 99]. Using FXS patient derived iPSCs, we can not only study human-specific pathways dysregulated in FXS patient cells, but also understand how *FMR1* gene silencing induced epigenetic changes affect other cellular processes beyond FMRP deficiency.

#### Neuronal differentiation of FXS hPSCs

Several studies using FXS iPSCs, FXS hESCs, and isogenic *FMR1* gene deleted (*FMR1*-KO) hPSCs have shown that FXS dorsal forebrain neural progenitor cells (NPCs) have increased proliferation but reduced neuronal differentiation [19, 100]. Raj et al. showed that FXS NPCs remain longer in the replication phase of the cell cycle, while control cells mainly stay outside of the cell cycle [100]. An early study shows that neurons differentiated from NPCs directly isolated from one 18-week gestation human postmortem FXS fetal tissue generated more TUJ1 + neurons (immature neurons) and these neurons had reduced neurite length compared to controls [101]. A study using FXS neurons also display reduced expression levels of TUJ1 [102], and FMRP deficient human neurons exhibit reduced complexity and shorter dendrites [51] and impaired axonal growth [103] compared to their controls. The results of these studies are similar to what has been found in *Fmr1*-KO animal models, supporting that FMRP plays an important role in neuronal maturation during neurodevelopment. Transcriptomic analysis using RNA sequencing and FMRP target identification using crosslinking immunoprecipitation followed by RNA sequencing (CLIP-seq) demonstrate that FXS and *FMR1*-KO NPCs have upregulation of genes involved in proliferation, but downregulation of genes related to neuronal differentiation, neuronal morphology, and synaptogenesis [19]. In addition, because hPSCs can be differentiated into different cell types, Li et al. were able to identify both unique and common FMRP targets in hPSC differentiated dorsal forebrain excitatory and ventral forebrain inhibitory NPCs and neurons, unveiling cell-type specific roles for FMRP [19]. Overall, a loss of FMRP leads to an aberrant cell cycle and dysfunction in neuronal maturation and differentiation in human neurons.

#### Electrical activity of human FXS neurons

A prominent phenotype found in hPSC derived FXS neurons is hyperexcitability [51, 104, 105]. Similar to EEG impairments and audiogenic seizures found in FXS animal models, the hyperexcitability of human FXS neurons might be a result of impaired neuronal maturation described above. On the other hand, deficits in calcium and sodium channels have been reported in human NPCs derived from FXS which can contribute to elevated neuronal activities [106]. It has been reported that human FXS neurons do not differ from control neurons in their mEPSC and/or mIPSC properties. However, FXS neurons fire more frequent and shorter action potentials [107]. Sodium channel blockers as well as calcium-activated potassium channel blockers have been used to rescue the elevated activities in human FXS neurons to control levels [106, 108]. Another study has discovered a decrease in GABAergic neurons in FXS organoids suggesting that excitatory/inhibitory imbalance may contribute to elevated activity, similar to what have been shown in mouse models [109–111]. Reactivation of *FMR1* expression in human FXS neurons rescues the hyperexcitability deficits [104]. Our group has also shown that human and mouse FXS neurons have mitochondrial deficits and hyperexcitability, and enhancing mitochondrial functions can rescue hyperexcitability [105].

#### hiPSC derived 3D brain organoids

Cultured human neurons can only be maintained for up to 12 weeks, limiting their application in the study of human embryonic brain development spanning 40 weeks. 3-dimensional cortical organoids derived from iPSCs can be cultured for more than a year and resemble gene expression patterns similar to mid-fetal development [112–114] making them a promising tool for studying human brain development and neurodevelopmental disorders. Organoids enable the measurement of cortical layer formation, synapse maturation, neuron migration, and changes in electrical activities across multiple time-points corresponding to early human development. Therefore, a number of studies have used organoids to study neurodevelopmental disorders, including Timothy syndrome [115, 116], Rett syndrome [117, 118], Down syndrome [119], autism [120], and FXS [111, 121].

The first FXS organoid study found that NPCs in organoids derived from three FXS patient iPSC lines had increased proliferation compared to their healthy controls at 28 days post-differentiation, which confirmed the findings obtained from their own [100] and other 2D studies [19, 100]. A second study used organoids from iPSCs that were CRISPR/Cas9 gene edited to knockout FMRP (*FMR1*-KO) and found that *FMR1* KO leads to overall bigger organoids from 50 to 100 days in culture which resembles the macrocephaly phenotype that has been reported in FXS patients [121]. They also observed more GFAP-expressing glial cells but did not observe significant changes in MAP2-positive neurons between FXS and control organoids [121]. However, the molecular mechanisms regulating these phenotypes were not investigated. The third study performed by Kang et al., 2021 did an in-depth analysis of FXS organoids and found deficits in cortical layer formation, synaptogenesis, electrical activity, and identified human-specific FMRP targets that can be pharmacologically manipulated to rescue these phenotypes [111]. However, they found that day 56 FXS organoids exhibited decreased proliferation along with an expanded cortical plate marked by layer 5 cortical marker CTIP2. It is possible that FXS cells have higher proliferation in early development and then rapidly transition to an accelerated neuronal differentiation near mid-fetal development, which must be confirmed. They also found that FXS organoids had an increase in synapse markers along with an increase in electrical activity. Their single-cell RNA sequencing (scRNA-seq) analysis of three pairs of FXS and control organoids show that FXS organoids were downregulated for genes important in neurogenesis, neuronal differentiation, morphogenesis, but were upregulated for protein translation and oxidative phosphorylation. However, they found that mGLuR5 inhibitors that typically rescue FXS phenotypes in mice did not rescue proliferation or synaptic activity in FXS organoids, but PI3K inhibitors did. This calls to the importance that FMRP might have species specific regulations and why human models should be used to confirm molecular pathways found in animal models for the identification of promising treatments.

#### Limitations of human stem cell models of FXS

The hPSC derived 2D or 3D models lack many of the cell-types and extracellular signals to guide the formation that reflect the brain anatomy found in vivo. Organoid models have emerged as a promising tool to study human brain development, however, standardized practices to ensure their rigor and reproducibility are still lacking and results from these models may be variable across laboratories [122]. In addition, limited studies using human post-mortem tissue indicate that cortical neurons in the FXS patients have higher density of immature spines [83, 84]. However, modeling spine development and maturation of human neurons are highly challenging due to the protracted developmental period of humans and limited maturation of human neurons in vitro. Furthermore, although functional assays can be performed in hPSC models, behavioral assays are not possible.

### Comparison among FXS models

Mechanistic investigations of human diseases require the use of experimental models. As famously stated by the renowned statistician George Box “All models are wrong but some are useful” [123]. Each FXS model has contributed significantly to our understanding of the role of FMRP in development and mechanism underlying FXS. In this review, we mainly discussed the drosophila, rodent, and human models of FXS. However, there are also other powerful models, including the *Fmr1*-KO zebrafish model [124], the chicken *ex ovo Fmr1* knockdown model [125], and the nonhuman primate ex vivo brain slice with *FMR1* knockdown model [51, 105]. None of these experimental models are perfect, but they recapitulate certain aspects of FMRP functions and FXS pathogenesis. The similarities and complementary features among these experimental models are driving the field forward to a unified understanding of the mechanisms of FXS.

**Fig. 1:**
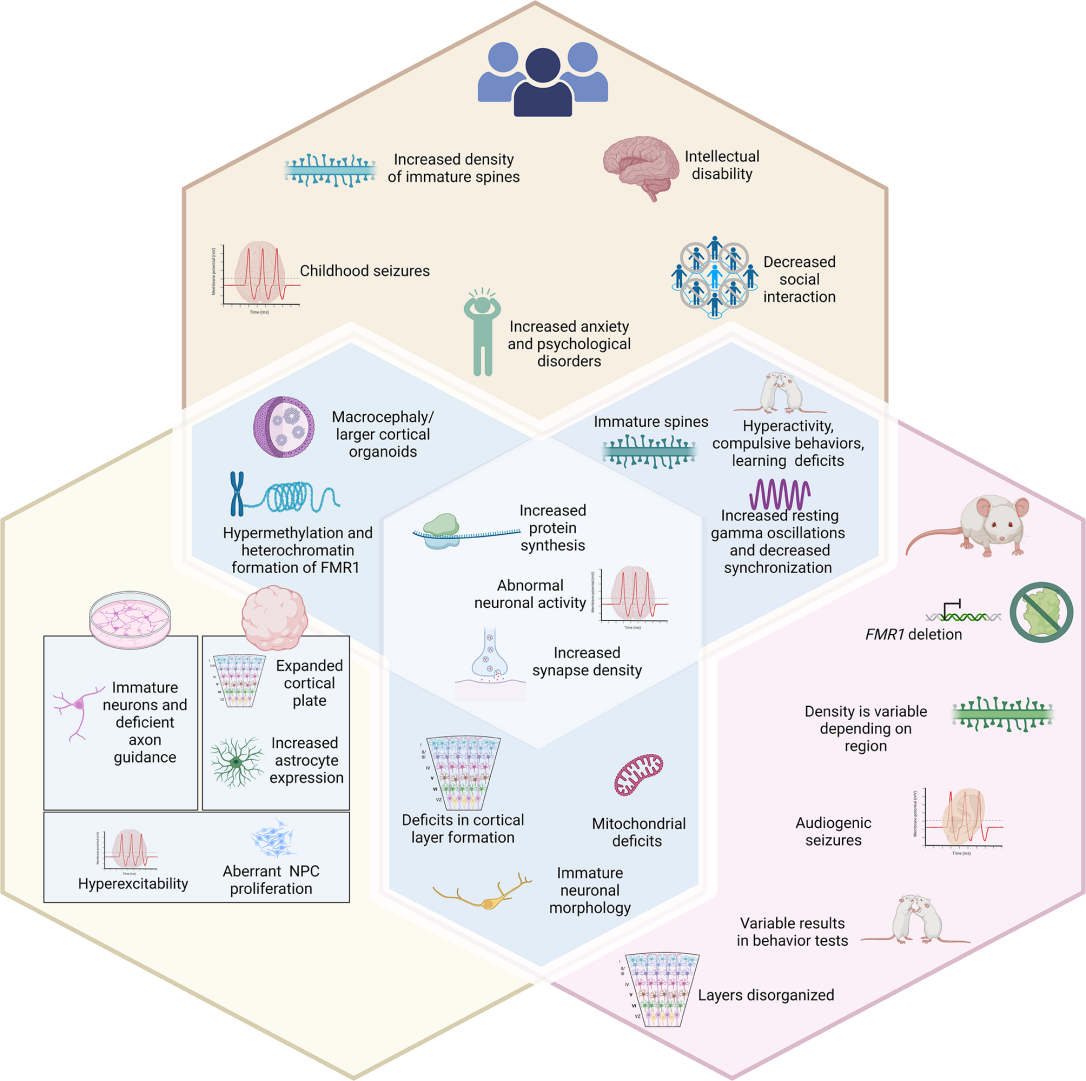
Comparison of phenotypes of FXS patients to those observed in *in vitro*. FXS human stem cell models and mouse FXS models. The symptoms in FXS patients **(top hexagon)** range from mild to severe. Some characteristics of FXS in patients overlap with autistic features such as intellectual disability, increased anxiety, decreased social interaction, and seizures during a young age. In pursuit of understanding the biological pathways behind FXS symptomology, some of these phenotypes have been successfully replicated in human pluripotent stem cells (hPSC) in vitro **(bottom left hexagon)** and mouse models of FXS **(bottom right hexagon).** The shared phenotypes include increased protein synthesis, abnormal neuronal activity, and increased synapse density. There are also some features that are unique to each model. In hPSC in vitro models, a prominent phenotype includes hyperexcitability and disruptions in NPC proliferation in cortical neurons and organoids. While in mouse models audiogenic seizures, immature spines, and disorganization in cortical layer formation have been observed. A main advantage to using hPSC in vitro models is that these neurons can be derived from FXS patient iPSCs that contain the epigenetic silencing of *FMR1* not found in mouse models allowing for more comprehensive biochemical analyses. However, in mouse models, behavior that can be correlated to FXS patients can be studied as has been done when assessing hyperactivity, compulsive behaviors, learning and memory deficits, as well as EEG properties. Overall, complementary model systems are needed to fully understand FXS. Please see Table S1 for a complete list and references. Figure created in BioRender.com.

Some of the major similarities among all the models of FXS that are comparable to patient data include increased protein synthesis, impaired neuronal activities, abnormal neuronal morphology, and altered synapse density [23] (Fig. 1). The rodent and drosophila models have been imperative to understand how FMRP deficiency influences behavior. Despite some variabilities among reports published by different laboratories, overall, both models have demonstrated that FMRP-deficiency leads to increased locomotor activity, increased repetitive behaviors, decreased social interaction, aberrant circadian rhythm, and impaired learning ability that match to that of human patient data. Data across the rodent, drosophila models, and human stem cell in vitro models have shown a consistent impairment in dendritic branching and synaptogenesis of neurons, altered neuronal activity, and defective calcium signaling [89]. Further, elevated resting gamma oscillations have been found in rodent models of FXS which matches those observed in human FXS individuals. These convergent phenotypes among FXS models suggest that FMRP may evolutionarily play similar roles in neuronal development across different species.

A major advantage of using animal models is that these models allow us to investigate FMRP and FXS in vivo and in intact brains. For example, some of the phenotypes such as impairments in neuronal activities, dendritic morphology, and synaptogenesis have been found to be brain region-specific, suggesting that FMRP may have differential regulatory roles in different brain anatomical regions [23]. Although cell type-specific FMRP targets have been identified in hPSC differentiated dorsal forebrain excitatory neurons and ventral forebrain inhibitory neurons [19], the investigation of brain-region specific role of FMRP is significantly more difficult to perform in human *in-vitro* stem cell models. Recent advancement of different region-specific organoids may help in this regard. However, organoids are still relatively immature and do not have the complex neural network present in an in vivo model.

Despite their limitations, hPSC models provide a number of advantages for studying FXS, including that we can assess the human-specific role of FMRP at different stages of development and in specific cell types [105, 111]. Human PSC models also allow us to investigate the timing and mechanism of *FMR1* gene silencing which cannot be modeled in animal models. Several recent advancements in the stem cell field will further enhance the use of human models in FXS. For example, dorsal forebrain and ventral subpallial assembloids can be used to study the E/I imbalance in FXS and compare in vivo data obtained from animal models and human EEG studies. However, only early fetal development can be effectively studied in human PSC models of FXS. Future development of bioengineering techniques for vascularized organoids and coculture techniques of organoids with glial cells can pave the way to understand the molecular mechanisms of FXS at late developmental time-points. Chimera models such as xeno-transplanted organoids in rodents may also be useful to understand how human FXS cells develop and function in an *in-vivo* environment.

## Conclusion

The FXS field has benefited from having a wide variety of experimental models with both convergent and complementary features. Given the complexity of FXS and human developmental and psychiatric disorders in general, it is essential to validate the observations obtained from one model in at least another model. Therefore, a combination of animal and human models will further help understand the pathogenesis of FXS and improve the development of effective treatments.

### Electronic supplementary material

Below is the link to the electronic supplementary material.

**Supplementary Table S1**. Summary of phenotypes found in Fragile X Syndrome patients and the drosophila, mouse, and human *in vitro* models of Fragile X Syndrome

## Data Availability

Data and materials are available through contacting the corresponding author: Xinyu Zhao (xinyu.zhao@wisc.edu).

## Abbreviations

ACC: anterior cingulate cortex
AMPA: α-amino-3-hydroxy-5-methyl-4-isoxazolepropionic acid receptor
cAMP: Cyclic adenosine monophosphate
cKO: Conditional knockout
cON: Conditional restoration
CLIP-seq: Crosslinking with immunoprecipitation sequencing
CTIP2: COUP-TF-interacting protein 2
dFmr1: Drosophila Fmr1
EEG: Electrocephalogram
FMR1: Fragile X messenger ribonucleoprotein 1
FMRP: Fragile X messenger ribonucleoprotein
FXS: Fragile X syndrome
FXTAS: Fragile X-associated tremor/ataxia syndrome
FXPOI: Fragile-X-associated primary ovarian insufficiency
GFAP: Glial fibrillary acidic protein
GSK3β: Glycogen synthase kinase-3 beta
hESCs: Human embryonic stem cells
hPSCs: human pluripotent stem cells
iPSCs: Induced pluripotent stem cells
KH: K homology
KO: Knockout
LTP: Long-term potentiation
MAP1B: Microtubule associated protein 1B
pre-mRNP: Pre-messenger ribonucleoprotein
sc-RNAseq: Single cell RNA sequencing
